# ROS-responsive GelMA/L-Arginine hydrogel with locally sustained NO release for accelerated skin wound healing

**DOI:** 10.3389/fphar.2026.1970915

**Published:** 2026-10-26

**Authors:** Yi Chen, Dongdong Wu, Haiyan Wu, Guojun Yu, Weixi Yang, Qing Wang, Weiyu Luo, Jing Wang

**Affiliations:** Burn and Plastic Surgery Department, The Affiliated Huaian No.1 People’s Hospital of Nanjing Medical University, Huaian, Jiangsu, China

**Keywords:** angiogenesis, hydrogel, macrophage polarization, ROS-responsive, skin wound healing

## Abstract

Nitric oxide (NO) plays crucial regulatory roles in accelerating cutaneous wound repair via modulating angiogenesis and macrophage polarization. Herein, a reactive oxygen species (ROS)-responsive GelMA/L-arginine (L-Arg) composite hydrogel was fabricated through 405-nm photocrosslinking for localized NO delivery and high-efficiency wound treatment. Physicochemical characterizations verified that L-Arg incorporation maintained the favorable moldability and interconnected porous microstructure of pristine GelMA hydrogel, and enabled sustained ROS-triggered NO release under oxidative microenvironment. *In vivo* full-thickness skin defect rat models demonstrated that the GelMA/L-Arg hydrogel markedly expedited wound closure, achieving a healing rate of 93.7% at day 14, and facilitated ordered re-epithelialization with a collagen area fraction of 64.6%. Further immunofluorescence results confirmed that the hydrogel improved regenerative quality by promoting mature angiogenesis with a vessel density of 116/mm^2^ and switching macrophages toward anti-inflammatory M2 phenotype. Systemic biosafety evaluation via major organ histological staining validated favorable biocompatibility without obvious organ toxicity. In conclusion, this multifunctional NO-releasing GelMA/L-Arg hydrogel represents a promising candidate for clinical wound dressing applications.

## Introduction

The skin serves as the primary physical barrier against dehydration, trauma and microbial invasion, and is critical for bodily homeostasis (Mao et al., 2021). Severe or impaired skin wounds compromise skin protective functions, harm patients’ physical and mental health, and impose a heavy burden on the healthcare system (Kurian et al., 2024). Conventional therapeutic strategies for skin wounds, including surgical suturing and skin grafting, still face inherent bottlenecks in clinical application, which are mainly limited by scarce donor resources and inevitable secondary tissue injury (Liu et al., 2026). To address these shortcomings, a variety of improved wound dressings represented by films, nanofibrous membranes and hydrogels have been extensively developed to accelerate wound repair and tissue regeneration (Zhang et al., 2021a; Qiao et al., 2026; Zhang et al., 2021b).

**SCHEME 1:**
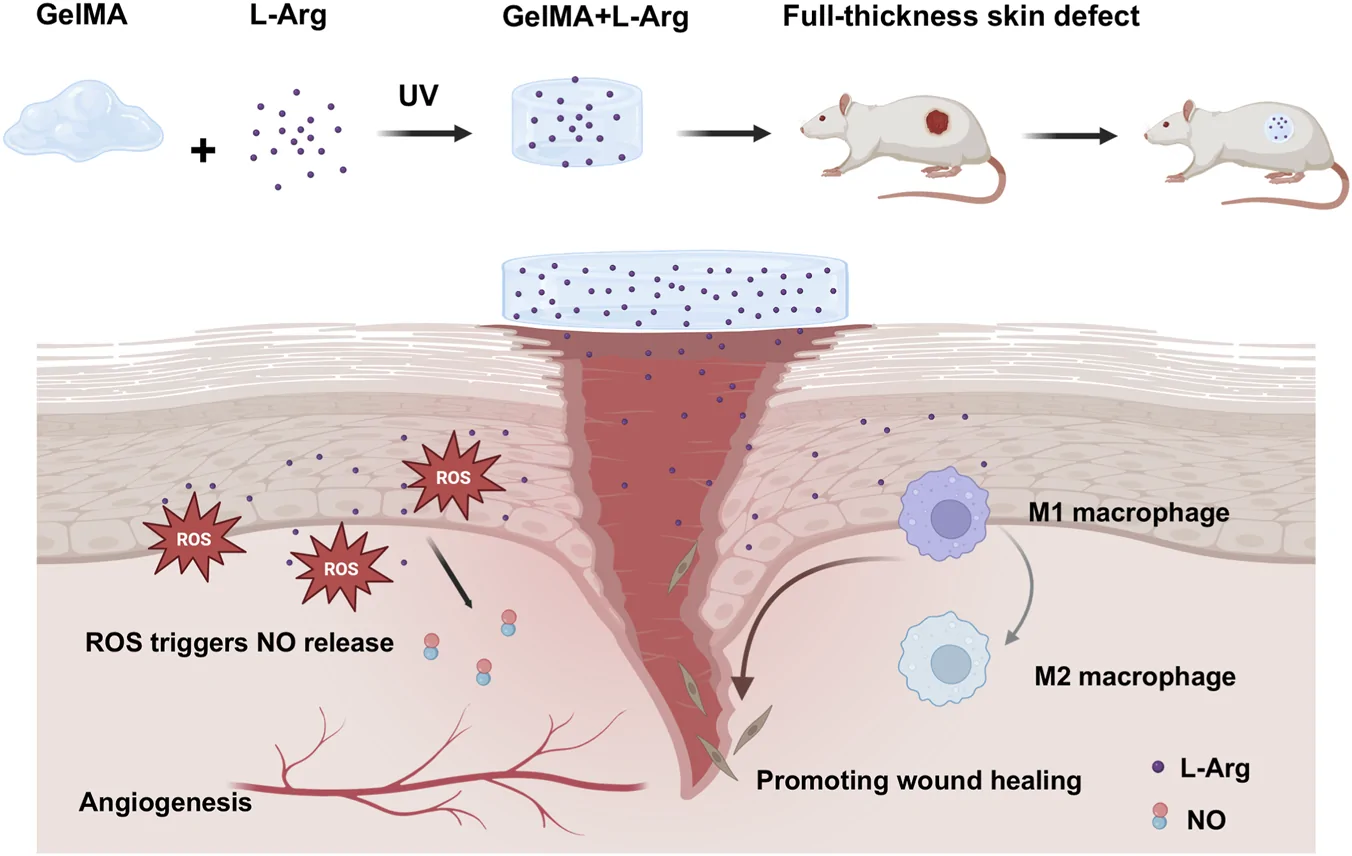
Schematic illustration of the ROS-responsive GelMA/L-Arg hydrogel for accelerated full-thickness skin wound healing.

Among these emerging dressing materials, hydrogels are regarded as the most promising option. Benefiting from their high-water content and unique three-dimensional network microstructure, hydrogels are capable of constructing a moist microenvironment that closely mimics the natural extracellular matrix (ECM), thereby providing favorable physiological conditions for wound healing (Qiao et al., 2026; Richbourg et al., 2024). As a typical natural biopolymer, gelatin is derived from collagen. Its derivative, gelatin methacryloyl (GelMA), combines the intrinsic advantages of gelatin with tunable physicochemical properties, and exhibits outstanding biocompatibility and biodegradability (Kurian et al., 2022). Accordingly, GelMA has become a versatile and high-potential biomaterial for a wide range of biomedical applications.

L-arginine (L-Arg) serves as a crucial modulator throughout wound repair. *In vivo*, L-Arg is catalyzed separately by nitric oxide synthase (NOS) and arginase, yielding nitric oxide (NO) and ornithine (Hristina et al., 2014). Moreover, the overproduction of reactive oxygen species (ROS) within the wound microenvironment can also convert L-Arg to NO (Wan et al., 2019). Additionally, L-Arg-derived NO exerts prominent antibacterial, anti-inflammatory, and pro-angiogenic effects (Wa et al., 2023).

During the inflammatory stage of wound healing, NO regulates leukocyte chemotaxis and the secretion of inflammatory mediators. It promotes angiogenesis via vasodilation and by stimulating endothelial cell proliferation, migration and differentiation. In the proliferative phase, NO enhances fibroblast migration and proliferation, thereby accelerating collagen synthesis and deposition at wound sites. Moreover, NO modulates keratinocyte growth and epidermal regeneration, and also possesses bactericidal capacity to inhibit and eradicate pathogens (Champeau et al., 2018; Xia et al., 2025). Meanwhile, ornithine acts as a precursor for proline synthesis, and further facilitates wound repair by serving as an essential substrate for collagen production (Ling et al., 2021). However, oral administration of L-Arg exhibits limited therapeutic efficiency, as it cannot achieve effective local accumulation at the wound site (He et al., 2014).

In this study, we fabricated a NO‐releasing hydrogel wound dressing using GelMA and L-Arg. The therapeutic effects of the prepared dressing were evaluated using a full-thickness skin defect rat model. The results demonstrated that the hydrogel possesses excellent anti-inflammatory activity, promotes angiogenesis, and accelerates skin wound healing. This work highlights that the GelMA/L-Arg hydrogel holds great potential as an innovative controlled NO-release platform for efficient wound therapy (Scheme 1).

## Materials and methods

### Preparation of GelMA/L-Arg hydrogel

GelMA monomer and lithium pherryl‐2,4,5‐trimethylbenzoylphosphinate (LAP) were purchased from Engineering for Life (China). GelMA hydrogel was prepared as previously described (Wei et al., 2024). Briefly, a 10% (w/v) GelMA working solution was prepared by dissolving the monomer in deionized water supplemented with 0.25% (w/v) LAP. For the GelMA/L-Arg hydrogel, 0.05 g of L-Arg (Sigma-Aldrich, United States) was added to 10 mL of the pre-crosslinked solution under constant stirring. Then, the solutions were sterilized via filtration through a 0.22 µm filter, transferred into round molds (2 cm in diameter), and subjected to 405-nm photocrosslinking at a working distance of 3 cm to yield uniform disk-shaped hydrogels.

### Characterization of GelMA/L-Arg hydrogel

To characterize the hydrogel microstructure, samples were first freeze‐dried overnight. The dried hydrogels were mounted on conductive carbon tape and sputter-coated with a thin layer of gold for 60 s through an Emscope SC500A splutter coater. Cross-sections of the dried sample were observed and photographed with a scanning electron microscope (SEM, ZEISS Gemany) at an accelerating voltage of 5–10 kV. The functional groups of the hydrogels were characterized using a Bruker Tensor II FTIR spectrometer over a scan range of 4,000 to 400 cm^−1^.

NO release was measured using the Griess assay kit (Beyotime, China). Simply, 50 μM, 100 μM and 200 μM of H_2_O_2_ was added to the hydrogel to react at 37 °C for different time (0, 0.5, 1, 3, 6, 12, 24 h), respectively. The supernatants were collected and measured in accordance to the manufacturer’s manual. Finally, the curves of NO concentration versus reaction time were plotted.

To characterize the swelling behavior of GelMA hydrogels, samples were immersed in pH 7.4 PBS within sealed tubes at 37 °C to simulate the physiological environment of wound exudate. At predetermined time intervals, hydrogel specimens were retrieved, and surface residual PBS was gently blotted with filter paper prior to weighing. The swelling ratio was calculated via the equation below:

$$\text{Swelling ratio }\left(\%\right)=\left(W_{t}-W_{0}\right)/W_{0}\times 100\%$$

where W_t_ represents the hydrogel weight at a designated swelling time point, and W_0_ corresponds to the initial weight of GelMA hydrogels.

To assess the degradable behavior of GelMA hydrogels, all samples were first freeze-dried and weighed individually to obtain the initial weight W_0_. The hydrogel bulk was submerged in sterile PBS (pH 7.4), and the tubes were wrapped with parafilm for tight sealing. All samples underwent incubation at 37 °C. At scheduled time points, specimens were taken out and washed with ultrapure water. Subsequent lyophilization was performed, followed by weighing to record the residual mass W_t_. The degradation rate of each group was quantified via the equation below:

$$\text{Degradation ratio }\left(\%\right)=\left(W_{0}-W_{t}\right)/W_{0}\times 100\%$$

### Cell culture and treatment

RAW 264.7 cells were cultured in DMEM (Hyclone) supplemented with 10% FBS, at 37 °C in the presence of 5% CO_2_, with daily medium change and passaging at 80%–90% confluence. RAW 264.7 cells were divided into three groups: LPS (200 ng/mL; Sigma)+PBS, LPS + GelMA and LPS + GelMA/LArg, and all groups were cultured in DMEM for 24 h.

### Full-thickness skin defect model and wound treatment

All animal experiments in this study were approved by the Experimental Animal Research Committee of Nanjing Medical University. The wound healing efficacy of the GelMA/L-Arg hydrogel was evaluated using a full-thickness excisional skin wound model. Briefly, thirty 8-week-old male Sprague-Dawley rats were anesthetized with 2% inhaled isoflurane. The rats were randomly divided into three groups: Control, GelMA and GelMA + L-Arg. The dorsal skin was shaved and disinfected, and a 2 cm-diameter full-thickness round skin wound was created using a disposable biopsy punch. Hydrogel dressings were applied to the wounds, followed by a protective bandage to prevent dehydration and self-inflicted damage. After recovery from anesthesia, the rats were returned to their cage and monitored daily.

Images of wound sites were regularly captured on days 0, 3, 7, and 14 post-surgery without sacrificing the animals, and wound areas were blindly quantified using ImageJ software (NIH, United States). The residual wound area was calculated and presented as a relative percentage normalized to the initial wound area defined as 100% at day 0. The wound healing rate was calculated according to the following formula:

$$\text{Wound healing rate}=\left({\text{S}}_{\text{initial}}-{\text{S}}_{\text{current}}\right)/{\text{S}}_{\text{initial}}\times 100\%$$

Following imaging on day 14, the animals were euthanized, and skin tissues were harvested for subsequent analyses.

### ROS assay

Wound tissues were harvested from rats in each group on day 3 post-treatment for oxidative stress biomarker detection. The collected wound tissues were thoroughly rinsed with pre-cooled sterile phosphate-buffered saline (PBS) to remove residual blood and tissue exudates, followed by thorough mincing on ice. Subsequently, tissue homogenates were prepared and centrifuged at a low temperature and high speed to isolate the supernatant. After centrifugation, the clarified supernatants were carefully collected and preserved for subsequent biochemical quantification of oxidative stress indicators. The tissue levels of reduced glutathione (GSH) (BC1175, Solarbio, China), malondialdehyde (MDA) (BC0025, Solarbio, China), and hydrogen peroxide (H_2_O_2_) (S0038, Beyotime Biotechnology, China) were measured using commercial kits according to the manufacturers’ instructions. The absorbance of each sample at the corresponding characteristic wavelength was measured using a SpectraMax M4 microplate reader (Molecular Devices, United States) at the specified wavelengths, and data were calculated using SoftMax Pro software (Molecular Devices, United States).

### Histological analysis

To systematically evaluate the *in vivo* wound healing efficacy and histological regeneration characteristics of different treatment groups, full-thickness wound tissue samples were harvested at 2 weeks post-surgery. The collected tissue specimens were immediately fixed in 4% paraformaldehyde solution for 24 h to maintain tissue morphological integrity and prevent structural degradation. After sufficient fixation, the tissue samples were subjected to a gradient dehydration procedure using ethanol solutions with increasing concentrations, followed by transparent treatment with xylene and paraffin infiltration. The processed tissues were then embedded in paraffin blocks for stable long-term preservation and continuous sectioning. The paraffin-embedded tissue blocks were sliced into continuous sections with a uniform thickness of 5 μm using the Leica RM2125 RTS (Leica Biosystem, Germany). The obtained tissue sections were baked, dewaxed, and rehydrated according to standard histological protocols.

Subsequently, the prepared sections were subjected to routine hematoxylin and eosin (H&E) staining (G1120, Solarbio) and Masson’s trichrome staining (G1340, Solarbio) to observe the general histological morphology, epithelial regeneration status, dermal tissue reconstruction, and collagen deposition distribution of wound tissues. After staining, all sections were dehydrated again, transparentized, and sealed with neutral gum. High-resolution tissue images were captured under an optical microscope at consistent magnification for subsequent quantitative analysis. The epidermal thickness, dermal thickness, and collagen volume fraction of wound tissues in each group were statistically quantified and analyzed, referring to the mature quantitative method reported in previous studies (Wang et al., 2025).

### Immunofluorescence staining

Immunofluorescence staining was carried out to detect the expression of key biomarkers associated with angiogenesis and macrophage polarization in wound tissues. Paraffin-embedded wound sections were dewaxed, rehydrated, and subjected to antigen retrieval using citrate buffer (pH = 6.0). After rinsing with PBS, the sections were blocked with 5% BSA at room temperature for 1 h to eliminate non-specific staining.

The sections were then incubated overnight at 4 °C with diluted primary antibodies at optimal working concentrations, including anti-CD31 (ab64543, 1:200, Abcam, United Kingdom), anti-α‐SMA (19245, 1:100, Cell Signaling Technology, United States), anti-CD68 (41077, 1:200, Cell Signaling Technology, United States) and anti-CD206 (ab64693, 1:200, Abcam, United Kingdom). After thorough PBS washing, the sections were incubated with Goat Anti-Rabbit IgG 488 and Goat Anti-Mouse IgG 594 secondary antibodies (1:500, Abcam, United Kingdom) for 1 h at room temperature in the dark. Cell nuclei were counterstained with DAPI.

Fluorescence images were acquired with a fluorescence microscope. The positive area percentages of CD31, α-SMA, CD68 and CD206 were quantitatively analyzed via ImageJ software. The CD86^+^/CD206^+^ parameter was determined by dividing the percentage of CD86-positive cell rate by the percentage of CD206-positive cell rate in each group.

### Biosafety evaluation

To investigate the safety of hydrogels, 14 days post-surgery, the main organs of rats including heart, liver, spleen, lung, and kidney were collected for tissue embedding and HE staining, and normal rats were used as controls.

### Statistical analysis

All experimental data are expressed as mean ± standard deviation (SD). Statistical analyses were performed using GraphPad Prism 8.3.0 software. One-way analysis of variance (ANOVA) combined with Tukey’s *post hoc* test was adopted to evaluate statistical differences among groups. A value of p < 0.05 was defined as statistically significant.

## Results

### Preparation and characterization of GelMA/L-Arg hydrogel

The procedure for preparing GelMA + L-Arg hydrogel is shown in Figure 1a. The homogeneous precursor mixture was transferred into PDMS molds, followed by 405-nm photocrosslinking, to obtain uniform, disk-shaped hydrogel wound dressings with a diameter of 2 cm (Figure 1b). The images of GelMA and GelMA + L-Arg hydrogel are shown in Figure 1c. No significant differences in shape, size, or thickness were observed between the two groups, indicating that the incorporation of L-Arg did not interfere with the crosslinking process or compromise the structural integrity and formability of the GelMA matrix. The internal porous microstructure of the hydrogels was further analyzed by SEM (Figure 1d). Cross-sectional images revealed that both hydrogels possessed a highly porous, interconnected network architecture, a feature essential for nutrient diffusion and cell infiltration in tissue engineering applications.

**FIGURE 1:**
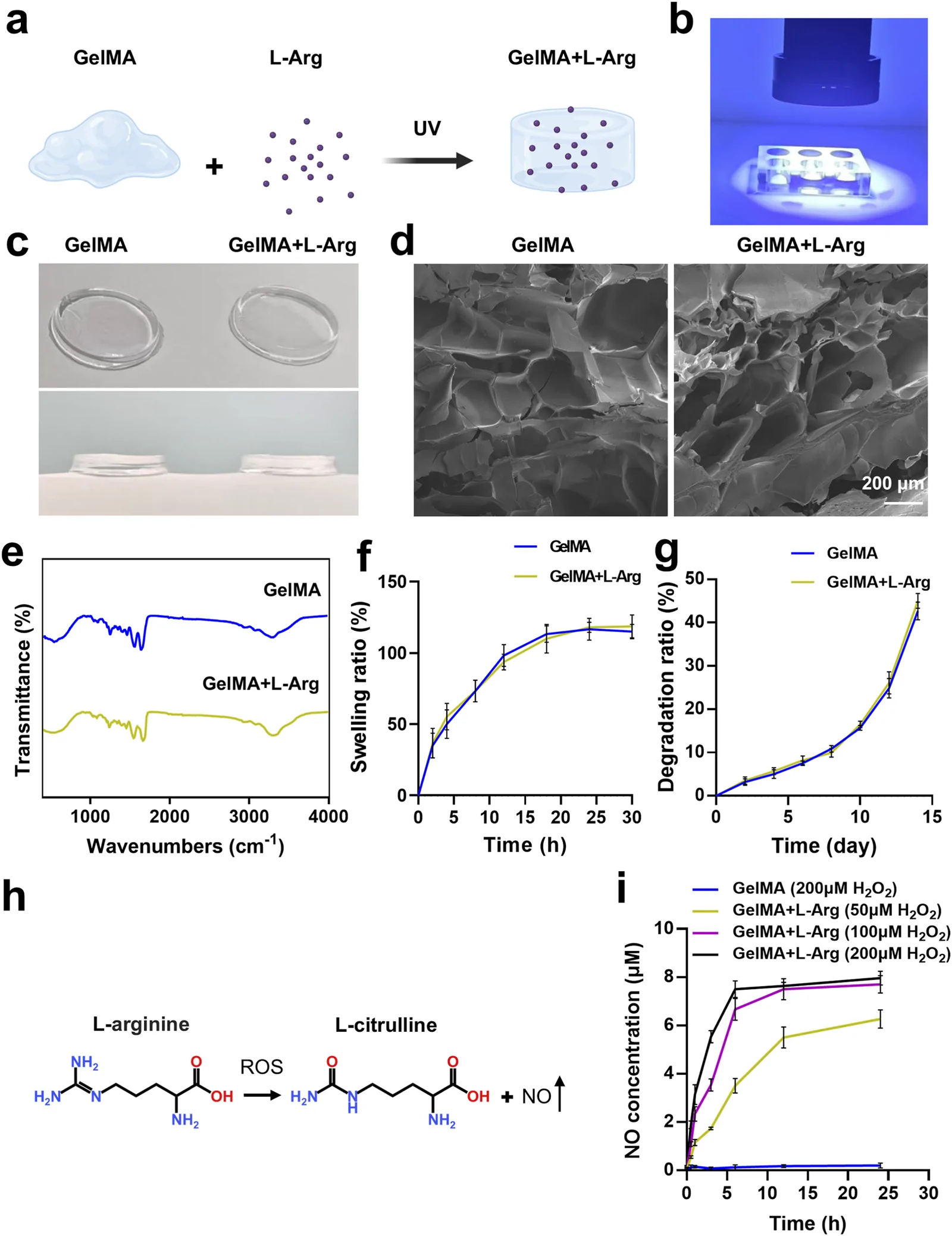
Fabrication and characterization of the ROS-responsive GelMA/L-Arg composite hydrogel. **(a)** Schematic illustration of the preparation process of GelMA/L-Arg hydrogel via UV crosslinking. **(b)** Photograph showing the UV crosslinking procedure of the hydrogel precursors. **(c)** Appearance of pure GelMA hydrogel and GelMA/L-Arg hydrogel. **(d)** Scanning electron microscopy (SEM) images of the lyophilized pure GelMA and GelMA/L-Arg hydrogels, revealing their interconnected porous microstructures. **(e)** Fourier transform infrared spectra of components of pure GelMA and GelMA/L-Arg hydrogels. **(f)** Swelling ratio of pure GelMA and GelMA/L-Arg hydrogels. **(g)** Degradation ratio of pure GelMA and GelMA/L-Arg hydrogels. **(h)** Chemical reaction scheme illustrating the non-enzymatic oxidation of L-Arg to NO triggered by ROS. **(i)** *In vitro* NO release profiles of pure GelMA and GelMA/L-Arg hydrogels under H_2_O_2_ over 24 h. Data are presented as mean ± SD (n = 3).

FTIR analysis revealed that the binding of L-Arg and GelMA was primarily achieved through physical bonds rather than chemical bonds (Figure 1e). The swelling profiles of pure GelMA and L-Arg-incorporated GelMA hydrogels are shown in Figure 1f. Both groups exhibited rapid water absorption in the initial stage, and their swelling ratios gradually plateaued after 20 h. No obvious difference in swelling capacity was observed between the two hydrogel systems, indicating the incorporation of L-Arg barely affected the water uptake property of GelMA networks. Figure 1g shows the hydrolytic degradation behavior of the two hydrogels over 14 days. The degradation ratio rose slowly in the early period and accelerated remarkably after day 10. Similarly, the two curves almost overlapped throughout the incubation period, confirming that L-Arg loading did not change the degradation performance of GelMA. The stable swelling and degradation profiles of GelMA/L-Arg hydrogel ensure sustained exudate absorption and matching tissue remodeling speed when applied as cutaneous wound dressings.

The ROS-triggered NO generation mechanism of the GelMA + L-Arg hydrogel is illustrated in Figure 1h. Under oxidative conditions, L-Arg undergoes a ROS-mediated oxidation reaction, and simultaneously releasing NO. The H_2_O_2_-triggered NO release profiles of hydrogels are displayed in Figure 1i. Virtually no was detected in pure GelMA treated with 200 μM H_2_O_2_, indicating L-Arg is required for NO production. All L-Arg-incorporated GelMA hydrogels displayed an initial rapid NO burst, followed by slow and sustained NO accumulation. The NO generation rate positively correlated with H_2_O_2_ concentration, with the 200 μM H_2_O_2_ group showing the highest NO production rate, followed by the 100 μM and 50 μM H_2_O_2_ groups.

The concentrations of three key oxidative stress markers, reduced glutathione (GSH), malondialdehyde (MDA), and hydrogen peroxide (H_2_O_2_), were quantitatively measured using commercial detection kits in rats 3 days post treatment (Supplementary Figure S1a–c). Compared with the Control group, the GelMA group showed no significant changes in the levels of GSH (p = 0.130), MDA (p = 0.272), and H_2_O_2_ (p = 0.189). In contrast, the GelMA + L-Arg treatment significantly increased intracellular GSH content (p < 0.001), while markedly reducing MDA and H_2_O_2_ levels (both p < 0.001). We further quantified the NO concentration in wound tissues (Supplementary Figure S1d). Consistent with the above findings, the NO level exhibited no statistical difference between the Control and GelMA groups (p = 0.427), whereas the GelMA + L-Arg group achieved a prominent elevation in tissue NO concentration (p < 0.001). These results suggest that the ROS-mediated oxidation of L-Arg is active in the rat full-thickness skin defect model, which boosts NO generation and alleviates oxidative stress at the wound site.

### GelMA/L-Arg hydrogel promotes wound healing

The therapeutic effect of the GelMA + L-Arg hydrogel on wound healing was evaluated using a full-thickness skin defect rat model (Figure 2a). Wound area was assessed at day 0, 3, 7, and 14, followed by histochemical analysis on day 14.

**FIGURE 2:**
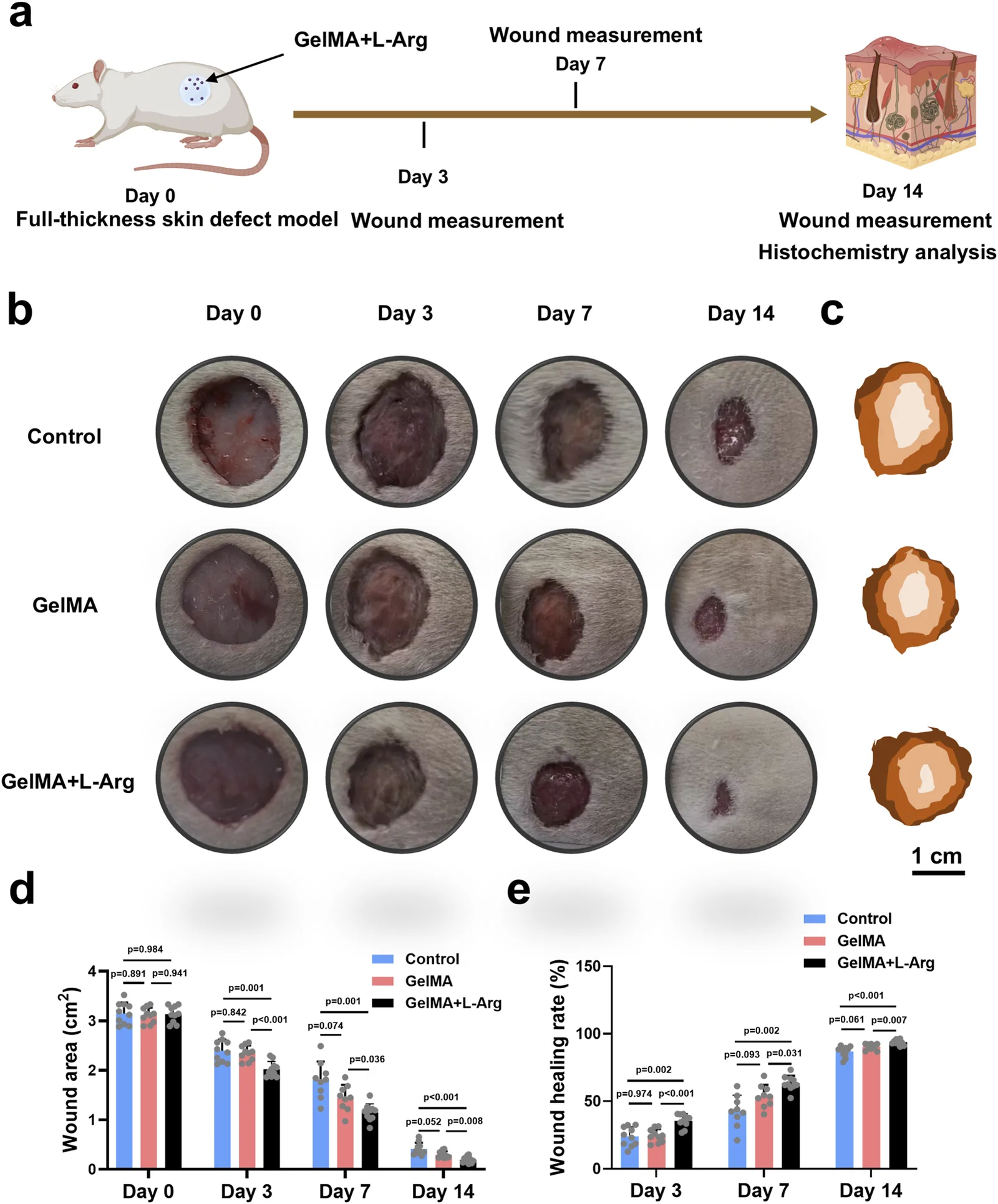
*In vivo* therapeutic efficacy of GelMA/L-Arg hydrogel in a rat full-thickness skin wound model. **(a)** Schematic illustration of the experimental timeline. **(b)** Representative images of wounds in the Control group, pure GelMA group, and GelMA + L-Arg group at the indicated time points. **(c)** Wound contour diagrams of each group showing the remaining unhealed areas. **(d)** Quantitative analysis of wound area changes over the 14-day observation period. **(e)** Quantitative analysis of wound healing rates in different groups. Data are presented as mean ± SD (n = 10 per group). Statistical significance was determined by one-way ANOVA followed by Tukey’s *post hoc* test.

Representative images of wounds treated with Control (untreated), pure GelMA, and GelMA + L-Arg hydrogels are shown in Figure 2b. At day 0, all groups exhibited similar initial wound sizes. Over the 14-day observation period, wounds in the GelMA + L-Arg group showed the most rapid reduction in size, with near-complete closure observed by day 14. In contrast, the Control and GelMA groups exhibited significantly slower healing, with larger residual wound areas remaining at the same time points. Schematic representations of the residual wounds at day 14 further confirmed the superior healing effect of the GelMA + L-Arg treatment (Figure 2c).

Quantitative analysis of wound area (Figure 2d) and wound healing rate (Figure 2e) further validated these observations. While all groups showed a gradual decrease in wound area over time, the GelMA + L-Arg group consistently displayed the smallest wound area and the highest healing rate at measured time points. At baseline (Day 0), there were no significant differences in wound size between the three groups, confirming the homogeneity of the initial injury. From Day 3 onward, the GelMA + L-Arg group showed a statistically significant reduction in wound area compared to the Control group (p < 0.05). By Day 14, the GelMA + L-Arg group achieved a significantly higher wound closure rate compared to both the Control and pure GelMA groups (p < 0.001 vs. Control; p = 0.007 vs. GelMA). Notably, the pure GelMA group also showed a moderate improvement in healing compared to the Control, but the effect was significantly less pronounced than that of the GelMA + L-Arg hydrogel.

These results demonstrate that the incorporation of L-Arg into the GelMA hydrogel significantly accelerates the *in vivo* wound healing process.

### Histological analysis of re-epithelialization and collagen deposition

To further evaluate the quality of wound regeneration, we performed histological analysis of skin tissue harvested on Day 14 post-treatment.

H&E staining (Figure 3a) was used to assess epidermal regeneration and dermal reconstruction. The Control group showed incomplete re-epithelialization with a discontinuous epidermal layer. In contrast, both hydrogel-treated groups exhibited enhanced epidermal coverage, with the GelMA + L-Arg group showing the most continuous, and well-organized epidermis, closely resembling native skin structure. Quantitative analysis of epidermal thickness (Figure 3c) confirmed the GelMA + L-Arg group (76.3 ± 18.5 μm) showed significantly lower epidermal thickness than the Control group (119.3 ± 28.0 μm, p < 0.001), and the GelMA group (109.4 ± 15.4 μm, p = 0.002), suggesting more physiological re-epithelialization. Dermal thickness (Figure 3d) was significantly decreased in both hydrogel groups compared to the Control (p < 0.001 for both), with the GelMA + L-Arg group showing a well-organized dermal architecture.

**FIGURE 3:**
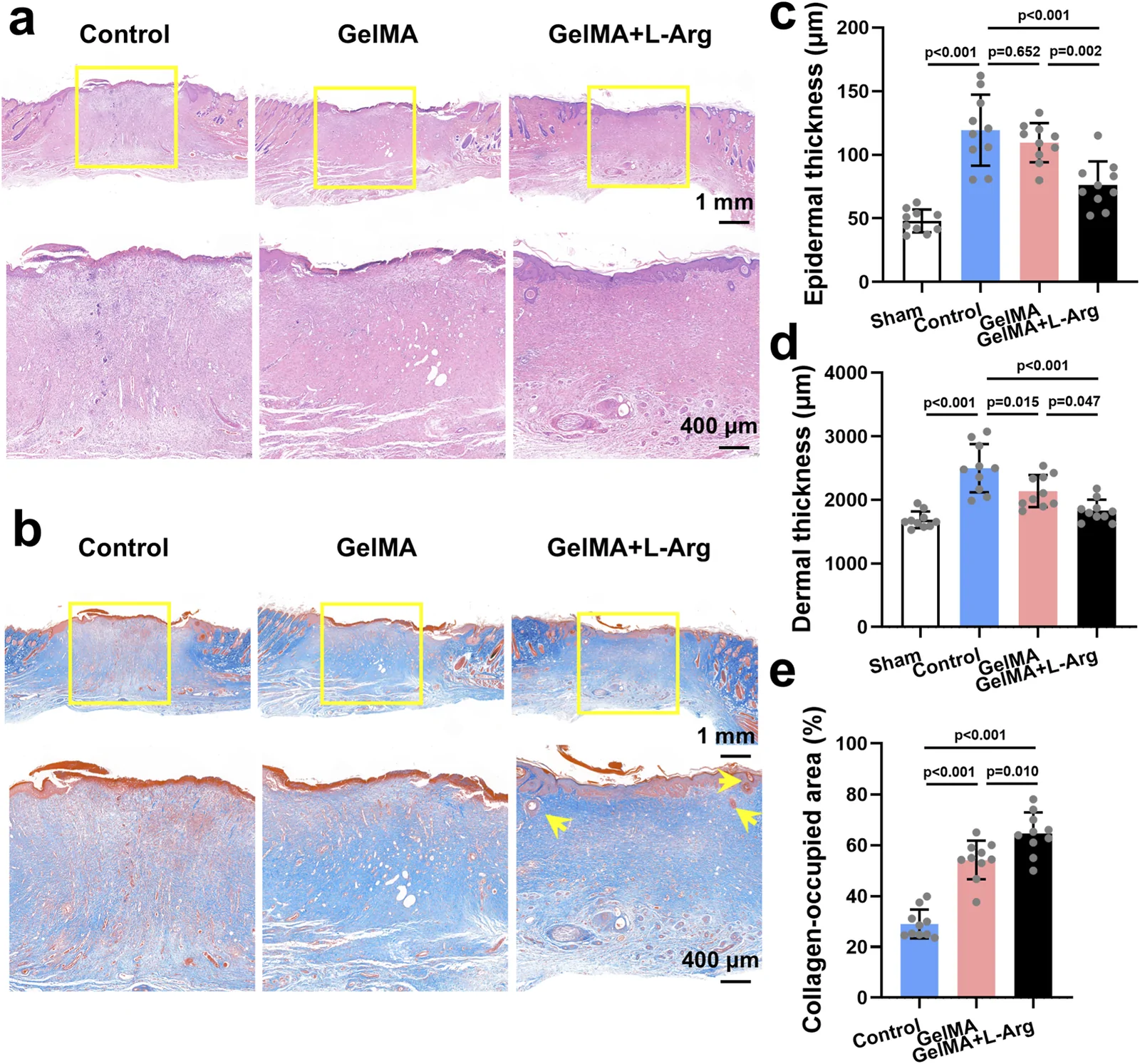
Histological evaluation of skin wound regeneration on day 14 post-surgery. **(a)** H&E staining of wound tissues from each group. The upper panels show low-magnification views, and the lower panels show high-magnification views of the yellow boxed areas. **(b)** Masson’s trichrome staining of wound tissues from the three groups. Yellow arrows indicate newly formed sebaceous glands and hair follicles. **(c)** Quantitative analysis of epidermal thickness in each group. **(d)** Quantitative analysis of dermal thickness in each group. **(e)** Quantitative analysis of collagen-occupied area percentage in the wound sites of the three treatment groups. All data are presented as mean ± SD (n = 10 per group). Statistical significance was determined by one-way ANOVA followed by Tukey’s *post hoc* test.

Masson’s trichrome staining (Figure 3b) was performed to evaluate collagen deposition and epithelial maturation. The Control group showed sparse, loosely packed collagen fibers. Both hydrogel treatments promoted more abundant and densely packed collagen deposition, with the GelMA + L-Arg group exhibiting the most extensive and organized collagen network, with fibers aligned parallel to the epidermal layer. Moreover, neogenic sebaceous glands and hair follicles were obvious in the GelMA + L-Arg group indicated by yellow arrows. Quantitative analysis (Figure 3e) revealed that the collagen-occupied area was significantly higher in the GelMA + L-Arg group (62.6% ± 8.3%) compared to both the Control (27.0% ± 5.7%, p < 0.001) and pure GelMA (54.2% ± 7.6%, p = 0.010) groups, indicating enhanced collagen synthesis and maturation.

Collectively, these histological results demonstrate that the GelMA + L-Arg hydrogel significantly promotes high-quality wound regeneration, characterized by accelerated re-epithelialization, decreased dermal thickness, and enhanced collagen deposition and organization.

## GelMA/L-Arg hydrogel promotes angiogenesis and M2 macrophage polarization in wound tissue

To assess the pro-angiogenic effect of the hydrogel treatments, we performed immunofluorescence staining for CD31 (endothelial cell marker, red) and α-SMA (smooth muscle cell marker, green) on day 14 wound tissue sections (Figure 4a). The formation of mature, functional blood vessels was evaluated by co-localization of these two markers. Representative immunofluorescence images showed that the Control group exhibited sparse vascular structures, with low expression of both CD31 and α-SMA. In contrast, the hydrogel-treated groups displayed more abundant and organized vascular networks. The GelMA + L-Arg group showed the highest density of blood vessels, with prominent co-localization of CD31 and α-SMA, indicating the formation of mature vessels.

**FIGURE 4:**
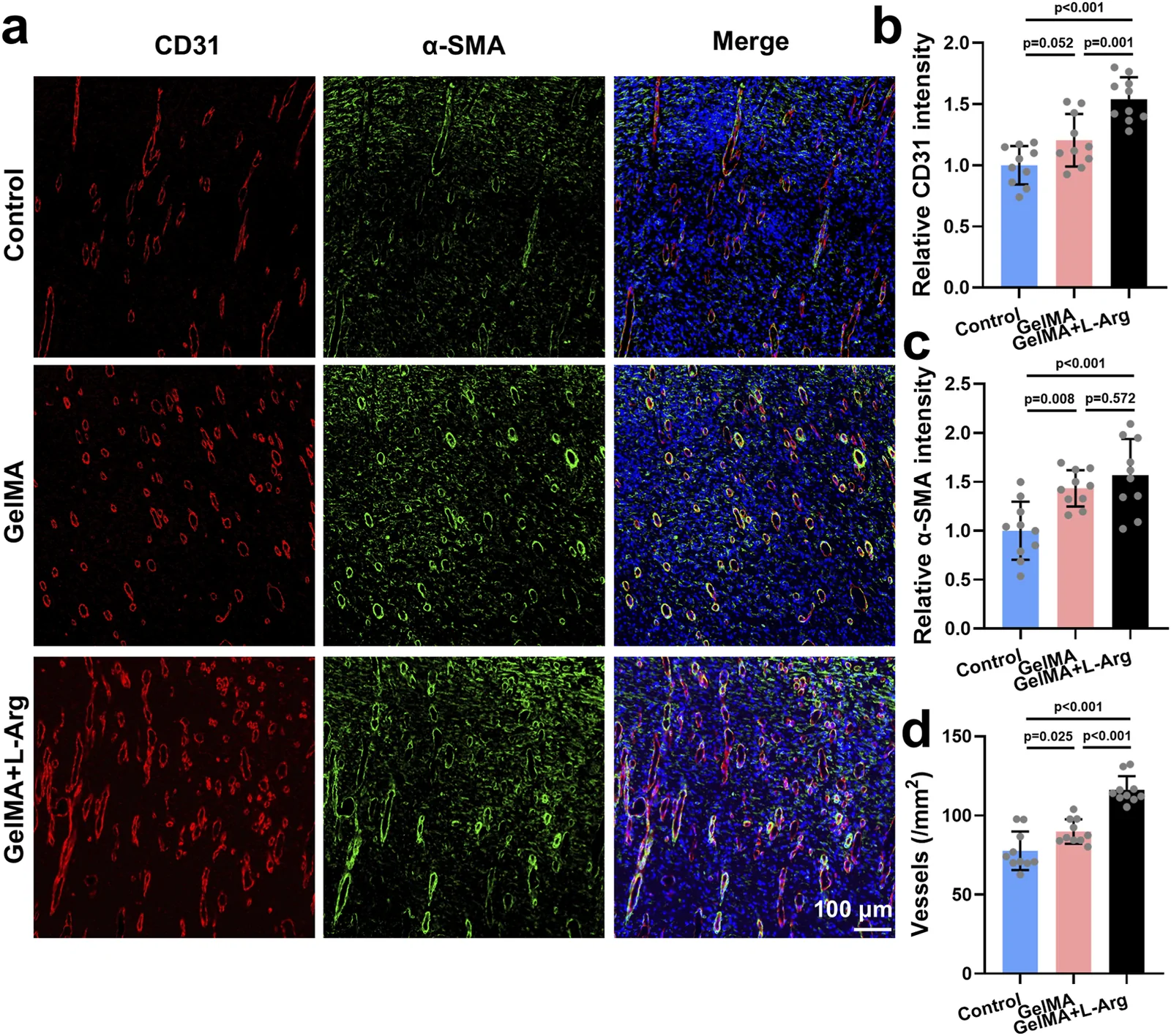
Immunofluorescence analysis of angiogenesis in regenerated skin tissues on day 14 post-wounding. **(a)** Representative immunofluorescence images of CD31 and α-SMA co-staining in wound sections from the Control, GelMA, and GelMA + L-Arg groups. **(b)** Quantitative analysis of relative CD31 fluorescence intensity. **(c)** Quantitative analysis of relative α-SMA fluorescence intensity. **(d)** Quantitative analysis of microvessel density, defined as the number of CD31-positive vessels per mm^2^ of regenerated tissue. All data are presented as mean ± SD (n = 10 per group). Statistical significance was determined by one-way ANOVA followed by Tukey’s *post hoc* test.

Quantitative analysis revealed that the relative CD31 intensity (Figure 4b) in the GelMA + L-Arg group was significantly higher than in both the Control (p < 0.001) and GelMA (p = 0.001) groups, while the GelMA group showed non-significant increase compared to the Control (p = 0.052). The GelMA + L-Arg group also exhibited the highest α-SMA intensity (Figure 4c), which was significantly elevated relative to the Control group (p < 0.001), with the GelMA group showing a moderate but significant increase compared to the Control (p = 0.008) but no significant difference from the GelMA + L-Arg group (p = 0.572). Furthermore, vessel density counting (Figure 4d) confirmed that the GelMA + L-Arg group had a significantly higher number of vessels than both the Control (p < 0.001) and pure GelMA (p < 0.001) groups, while the GelMA group also showed a significant improvement over the Control (p = 0.025).

These results demonstrate that both hydrogel treatments promote angiogenesis, but the GelMA + L-Arg hydrogel exerts a significantly enhanced pro-angiogenic effect, leading to the formation of more abundant and mature blood vessels.

To evaluate the immunomodulatory effect of the hydrogels in the wound microenvironment, immunofluorescence staining was performed for CD68 (a marker of total macrophages, red) and CD206 (a marker of anti-inflammatory M2 macrophages, green) at day 14 post-treatment (Figure 5a). While the Control group showed predominantly CD68-positive cells with sparse CD206 expression, both hydrogel-treated groups exhibited increased CD206 signal, with the GelMA + L-Arg group showing the most abundant and widespread CD206 staining. Quantitative analysis of the CD206^+^/CD68^+^ ratio (Figure 5b) confirmed these observations: the ratio was significantly higher in the GelMA + L-Arg group compared to both the Control (p < 0.001) and GelMA (p < 0.001) groups, and the GelMA group also showed a significantly elevated ratio relative to the Control (p = 0.010). These results demonstrate that the GelMA + L-Arg hydrogel strongly promotes the polarization of macrophages toward the anti-inflammatory, pro-healing M2 phenotype, creating a favorable immune microenvironment that likely contributes to the accelerated and improved quality of wound regeneration.

**FIGURE 5:**
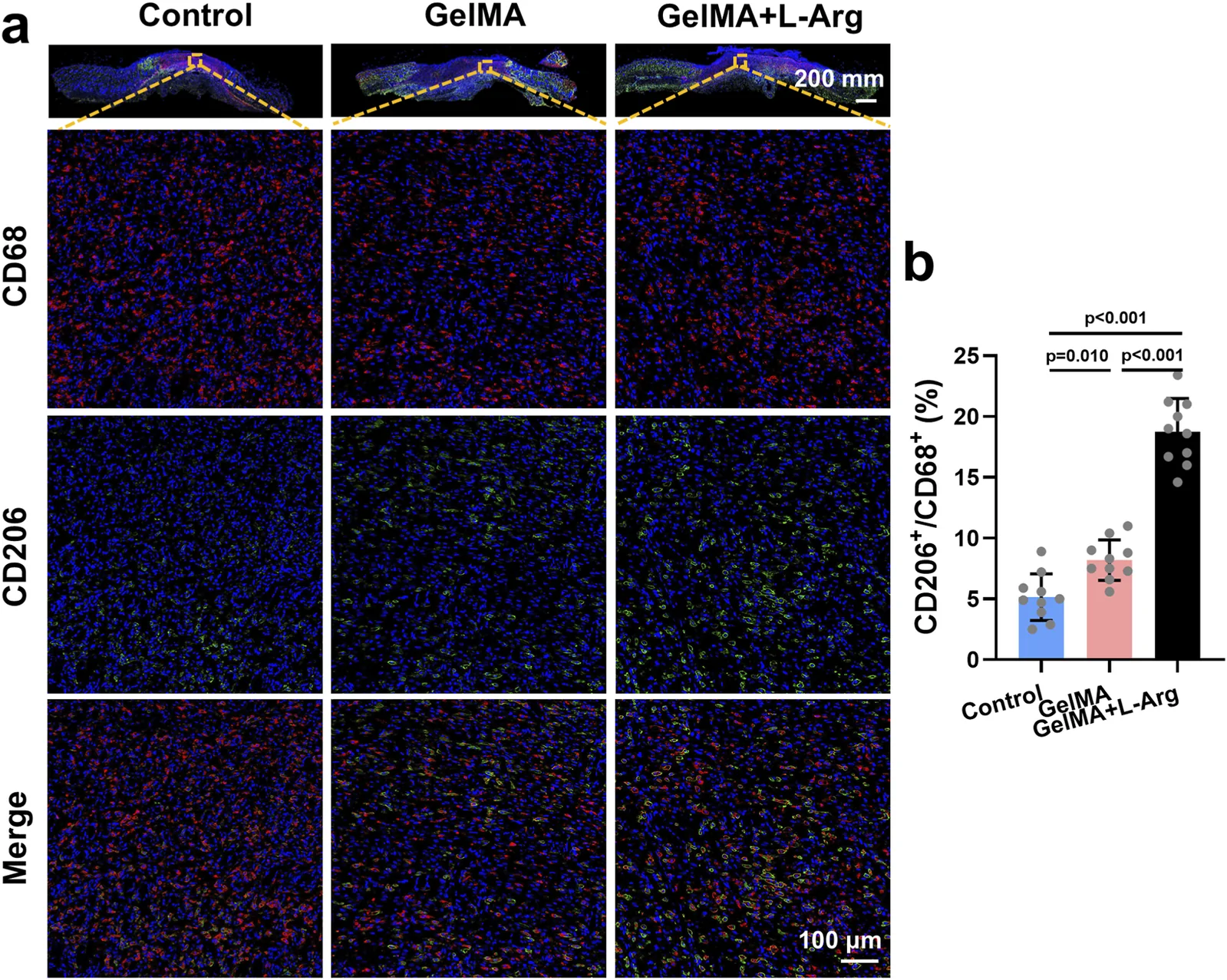
Immunofluorescence analysis of macrophage polarization in wound tissues on day 14 post-wounding. **(a)** Representative immunofluorescence images showing the expression of total macrophage marker CD68 (red) and M2 macrophage marker CD206 (green) in wound sections from the Control, GelMA, and GelMA + L-Arg groups. **(b)** Quantitative analysis of the ratio of CD206^+^ M2 macrophages to CD68^+^ macrophages in the wound sites. All data are presented as mean ± SD (n = 10 per group). Statistical significance was determined by one-way ANOVA followed by Tukey’s *post hoc* test.

To assess the regulatory effect of L-Arg-laden GelMA on LPS-triggered macrophage polarization, we performed immunofluorescence staining for M1 marker CD86 and M2 marker CD206 on RAW 264.7 cells (Figure 6a). The CD86^+^/CD206^+^ ratio reflects the relative balance between pro-inflammatory M1-type (CD86^+^) and anti-inflammatory M2-type (CD206^+^) macrophages. After LPS treatment, macrophages cultured with PBS or GelMA hydrogel were predominantly CD86-positive M1 cells with minimal CD206 expression, and their CD86^+^/CD206^+^ ratios were statistically indistinguishable (p = 0.852). However, the GelMA + L-Arg treatment remarkably downregulated CD86 and upregulated CD206 expression, driving macrophages toward M2 phenotype. Quantitative statistics of CD86^+^/CD206^+^ proportion (Figure 6b) revealed a significant reduction in the GelMA + L-Arg group relative to PBS and GelMA groups (p < 0.001). These results demonstrated that L-Arg loaded GelMA hydrogel exerts potent M2 polarization-promoting effects on LPS-activated macrophages.

**FIGURE 6:**
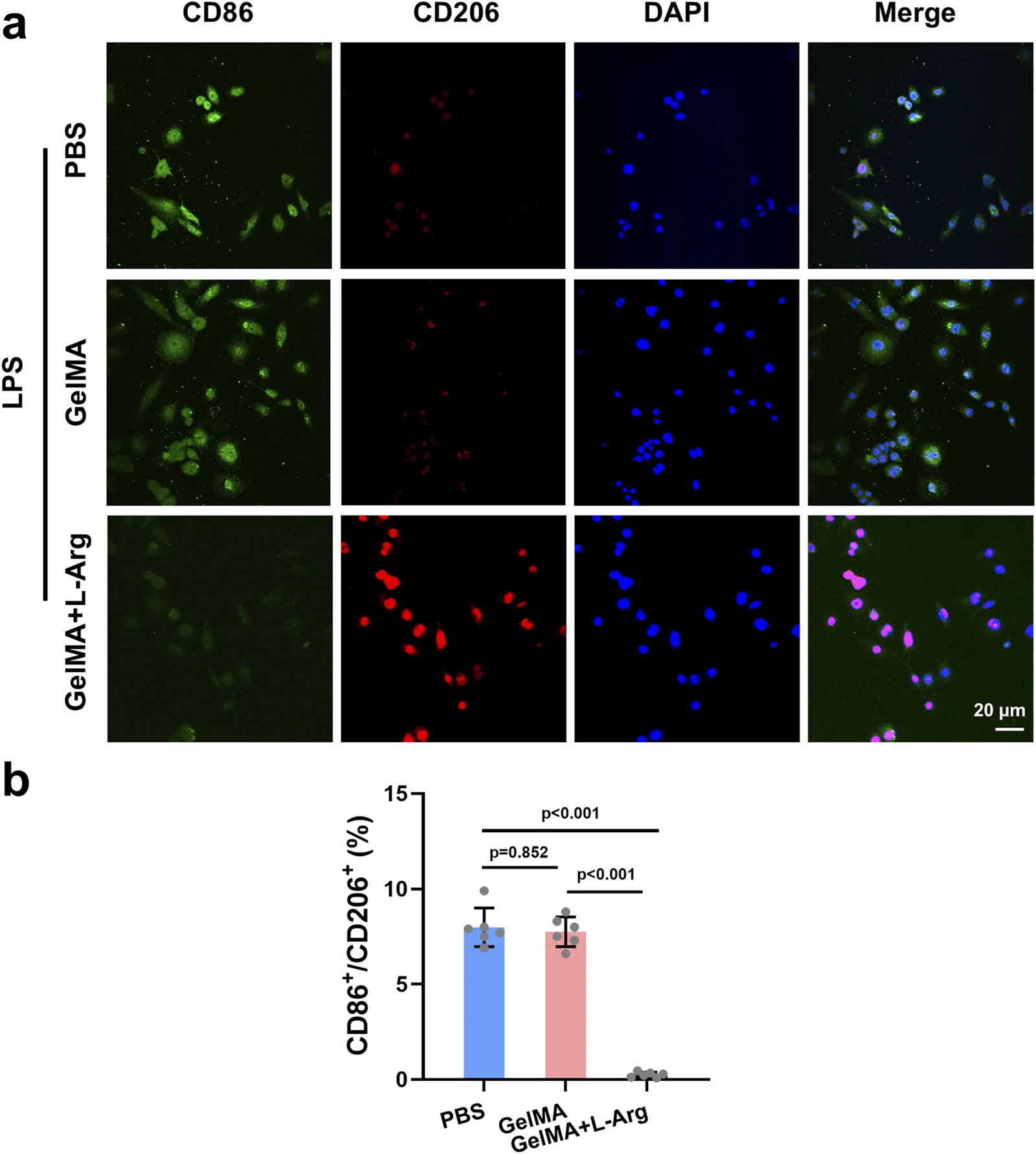
L-Arg-loaded GelMA hydrogel reverses LPS-induced M1 macrophage polarization. **(a)** Representative immunofluorescence images of macrophages stained for M1 marker CD86 (green), M2 marker CD206 (red), and nuclear dye DAPI (blue). **(b)** Quantitative statistics of CD86^+^/CD206^+^ cell ratio across three groups. All data are presented as mean ± SD (n = 6 per group). Statistical significance was determined by one-way ANOVA followed by Tukey’s *post hoc* test.

### *In vivo* biosafety evaluation of the hydrogels

To assess the systemic biocompatibility and potential toxicity of the hydrogel treatments, histological analysis of major organs (lung, liver, spleen, kidney, and heart) was performed via H&E staining at day 14 post-treatment (Figure 7). Representative images showed that all organs from the Control, GelMA, and GelMA + L-Arg groups exhibited normal tissue architecture with no signs of inflammation, necrosis, or pathological abnormalities. Specifically, the lung alveolar structure was intact, the liver showed normal hepatic lobule morphology, the splenic white and red pulp were well-organized, the renal tubules and glomeruli appeared healthy, and the myocardial fibers were arranged normally without evidence of damage.

**FIGURE 7:**
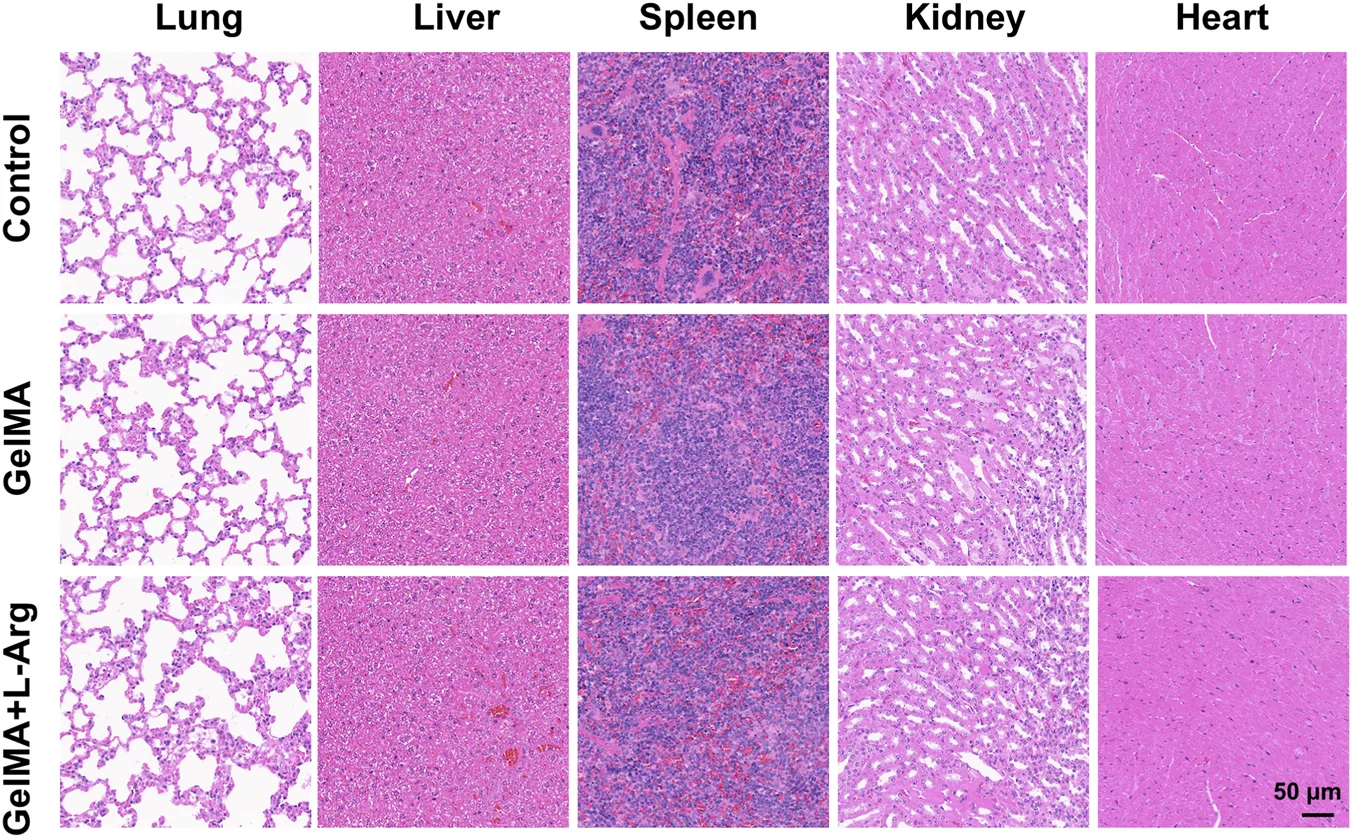
*In vivo* systemic biocompatibility evaluation of the GelMA/L-Arg hydrogel. H&E staining of major organs (lung, liver, spleen, kidney, and heart) harvested from rats in the Control, GelMA, and GelMA + L-Arg groups on day 14 post-wounding.

These results demonstrate that both the pure GelMA and GelMA/L-Arg hydrogels possess excellent *in vivo* biosafety, with no detectable systemic toxicity to major organs after topical application in the wound model.

## Discussion

Skin wound healing is a process involving sequential inflammation, angiogenesis, and tissue remodeling to restore skin tissue integrity (Eming et al., 2014). Developing advanced wound dressings for high-quality regenerative repair is critical for clinical wound treatment. Among diverse dressing candidates, GelMA hydrogel stands out owing to superior biocompatibility, biodegradability and photocrosslinkable processability derived from native gelatin (Yue et al., 2015). In this work, a ROS-responsive GelMA/L-Arg composite hydrogel was successfully fabricated. Incorporation of L-Arg did not compromise the moldability, structural integrity or porous interconnected microstructure of pristine GelMA hydrogel, which provides favorable conditions for cell infiltration, nutrient exchange and tissue ingrowth (Seyedsalehi et al., 2024).

Our hydrogel is fabricated via one-step visible light crosslinking, without nanomaterial doping, chemical modification of GelMA backbone, or multi-layer assembly. This greatly reduces preparation difficulty and batch-to-batch variation, and is compatible with large-scale production. NO generation is activated by intrinsic ROS in the wound microenvironment, without requiring external NIR laser irradiation. This avoids potential thermal damage to peri-wound tissues and simplifies clinical operation without auxiliary equipment. The system contains no inorganic nanoparticles, eliminating potential risks of long-term tissue retention and systemic toxicity. The simple composition and mild preparation conditions endow the system with broader application scenarios and lower production costs, making it more suitable for clinical translation as a routine wound dressing. We also acknowledge that the current system lacks anti-biofilm activity for severe infected ulcers, which will be a key direction for our future optimization.

Benefiting from the ROS-triggered reaction of L-Arg, the hydrogel achieves sustained NO release under oxidative microenvironments. As demonstrated in previous literature, among the ROS present in inflammatory wounds, H_2_O_2_ is the primary ROS that directly triggers nonenzymatic NO production from L-Arg. Superoxide anion (O_2_^•-^) cannot oxidize L-Arg to generate NO, but rapidly reacts with newly formed NO to produce peroxynitrite (ONOO-), thereby reducing NO bioavailability. Hydroxyl radicals (·OH) non-specifically degrade L-Arg and do not induce NO generation (Wa et al., 2023; Wu et al., 2026). Moreover, the nonenzymatic NO production from L-Arg triggered by H_2_O_2_ is pH-dependent. Weakly acidic conditions accelerate the oxidation of the guanidinium group of L-Arg and promote NO release, whereas strong alkaline environments suppress this reaction (Fan et al., 2017). The inflammatory wound microenvironment maintains near-neutral pH, which is suitable for the H_2_O_2_-mediated NO release of our hydrogel. *In vivo* detection on day 3 further verified this mechanism: the GelMA/L-Arg group showed significantly decreased endogenous H_2_O_2_ and increased NO in wound tissues (p < 0.001 vs. Control and GelMA), directly confirming that immobilized L-Arg consumed wound-derived H_2_O_2_ to generate NO *in situ*. Meanwhile, the hydrogel elevated GSH and reduced MDA levels, simultaneously ameliorating local oxidative stress.

*In vivo* full-thickness skin defect rat models confirmed the superior healing efficacy of GelMA/L-Arg hydrogel. Macroscopic observation and quantitative analysis demonstrated that the composite hydrogel significantly accelerated wound closure and reduced residual wound area compared with the blank control and pure GelMA groups within 14 days. Histological evaluation further verified improved regenerative quality in the GelMA/L-Arg group. The composite hydrogel promoted complete, ordered re-epithelialization and normalized dermal structure. Moreover, it markedly enhanced dense and ordered collagen deposition, facilitated extracellular matrix remodeling, and induced the regeneration of skin appendages, avoiding disorganized fibroplasia and enabling physiological wound regeneration.

Mechanistically, the excellent healing performance of GelMA/L-Arg hydrogel could be partially attributed to from L-Arg-mediated NO generation and its synergistic pro-angiogenic and immunomodulatory effects (Hristina et al., 2014; Wan et al., 2019). Immunofluorescence results revealed that the hydrogel significantly upregulated CD31 and α-SMA expression, increasing mature vascular density and promoting functional angiogenesis to support nutrient and oxygen supply for wound repair, consistent with the reported pro-proliferation effect of NO on endothelial cells (Champeau et al., 2018). In addition, GelMA/L-Arg hydrogel effectively modulated the wound immune microenvironment by elevating the CD206/CD68 ratio, thereby promoting M2 macrophage polarization to resolve local inflammation. Unlike systemic oral L-Arg with poor local enrichment at injury sites (Zhou et al., 2021), hydrogel immobilization realizes *in situ* retention of L-Arg and localized NO production directly targeting wound oxidative microenvironment, forming a positive regulatory loop to accelerate high-quality wound healing.

Notably, the therapeutic effect may arise from synergistic contributions of L-Arg’s nutritional function and ROS-responsive NO release. L-Arg serves as a precursor for collagen biosynthesis (Arribas-Lopez et al., 2021). Notably, even when NO production is inhibited by NOS inhibitors, L-Arg remains capable of enhancing net protein synthesis within skin wounds (Zhang et al., 2008). Meanwhile, locally generated NO promotes angiogenesis and drives M2 macrophage polarization. Together, these processes cooperatively accelerate high-quality wound regeneration. H&E staining of major organs confirmed the favorable *in vivo* biosafety of GelMA/L-Arg hydrogel, with no observable inflammation, necrosis or pathological abnormalities, supporting its potential clinical translatability. In summary, this study demonstrates that L-Arg-mediated sustained NO release efficiently enhances angiogenesis and optimizes macrophage polarization, thereby accelerating rapid and high-quality wound repair. Limitations of this work include the inability to distinguish the inherent bioactivity of L-Arg from ROS-triggered NO-mediated effects, insufficient exploration of downstream molecular signaling pathways, and the absence of long-term *in vivo* evaluation. Future research will further optimize hydrogel formulations and validate its therapeutic effects in large animal models to promote its clinical application as a high-performance wound dressing.

## Conclusion

Herein, a ROS-responsive GelMA/L-Arg composite hydrogel was successfully fabricated for skin wound therapy. The incorporation of L-Arg well preserved the superior moldability and interconnected porous microstructure of pristine GelMA hydrogel, while achieving targeted and sustained local NO release at wound sites. *In vivo* experiments confirmed that the optimized hydrogel significantly accelerated wound closure, facilitated re-epithelialization and ordered collagen deposition. Mechanistically, it promoted high-quality wound regeneration by enhancing angiogenesis and facilitating M2 macrophage polarization. With favorable *in vivo* biosafety, this GelMA/L-Arg hydrogel presents a promising localized therapeutic platform for clinical wound repair. Despite these promising results, several challenges remain. The precise spatiotemporal control of NO release requires further optimization to accommodate the dynamic demands of distinct wound-healing phases. In addition, the therapeutic effect on bacteria-infected wounds and chronic pathological wounds remains to be systematically verified.

## Data Availability

The original contributions presented in the study are included in the article/Supplementary Material, further inquiries can be directed to the corresponding author.
