# Supplementary material for: ROS-responsive GelMA/L-Arginine hydrogel with locally sustained NO release for accelerated skin wound healing
Source: Front Pharmacol. 2026 Oct 26;17:1970915. doi: 10.3389/fphar.2026.1970915 (PMC13746302; doi:10.3389/fphar.2026.1970915)
Supplement: Supplementary file 1 [file Supplementaryfile1.docx]

**Supplementary Materials**

*Figure S1. The concentrations of oxidative stress markers (a) GSH, (b) MDA, (c) H_2_O_2_ and (d) NO in the tissues 3 days post-surgery were quantitatively assessed using the detection kit. Data are presented as mean ± SD (n = 9 per group). Statistical significance was determined by one-way ANOVA followed by Tukey's post-hoc test.*
